# No aftereffect of motor duration production on auditory duration perception

**DOI:** 10.1038/s41598-026-59220-4

**Published:** 2026-06-23

**Authors:** Alessandro Mancari, Maria Concetta Morrone

**Affiliations:** 1 Department of Translational Research on New Technologies in Medicine and Surgery, University of Pisa, Pisa, Italy; 2 Department of Psychiatry, University of Oxford, Oxford, UK

**Keywords:** Time, Duration, Adaptation, Cross-modal adaptation, Neuroscience, Psychology, Psychology

## Abstract

Adaptation to stimulus duration causes a repulsive aftereffect on the perceived duration of subsequent stimuli. Most studies investigating duration adaptation have found that the effect is confined to the adapted modality, highlighting unimodal components of duration processing. Here, we use adaptation to test whether motor and auditory duration processing rely on partially shared neural mechanisms by looking for a transfer of the duration aftereffect between these modalities. We asked participants to estimate the perceived duration of auditory stimuli following auditory or motor adaptation. While we replicated the unimodal effects of auditory adaptation, we report that motor adaptation did not produce the typical repulsive aftereffect. We interpret this result as evidence that duration processing by the motor and auditory systems is based on independent mechanisms.

**Supplementary Information:**

The online version contains supplementary material available at https://doi.org/10.1038/s41598-026-59220-4.

## Introduction

We have a great ability to perceive the temporal duration of stimuli of different modalities, and similarly, to control the duration of our motor actions, such as a key press^1–5^. However, it is not clear to what extent the neural processing of temporal duration relies on independent modality-specific clocks whose perturbation would not affect time perception in other modalities.

Previous studies have shown that duration perception can be affected by sensory adaptation. For example, adaptation to high temporal frequency (20-Hz drifting gratings or flickering Gaussian blobs) causes a spatially localized compression of the perceived duration of visual stimuli, even when aftereffects on perceived frequency or speed have been annulled^6–8^. The same adaptation protocol also reduces apparent spatial and temporal numerosity^9^, suggesting that temporal frequency adaptation does not act directly on the representation of duration, but rather modulates the metric used by the visual system to represent space, time, and numerosity^10^.

Specifically for duration modulation, adaptation to repeated stimuli of constant duration induces repulsive aftereffects consistent with the existence of duration-tuned channels in vision and audition^11^. While most studies using this technique have emphasized the role of unimodal duration channels in both modalities^11–17^, a recent study investigated the cross-modal transfer of the duration aftereffect from vision to audition, and observed a partial transfer when auditory and visual stimuli are equated for perceived duration before adaptation^18^. Interestingly, also within the visual modality, shared mechanisms of duration adaptation across spatial locations in external coordinates can be revealed only when the stimuli have been equated for saliency^8^. Thus, duration adaptation has proven to be a sensitive technique to establish both independence and commonality of timing mechanisms across the senses.

Adaptation has also been applied to investigate temporal interactions between action and perception. The most studied is the perceived temporal separation between one’s own actions and their sensory consequences, which, after adaptation, can undergo a recalibration following exposure to an artificially increased delay of the sensory feedback. This, in turn, produces an aftereffect which can go as far as inverting the perceived temporal order of an action and its consequence^19–23^.

The effects of motor adaptation on the perception of stimulus duration have been little explored. This contrasts with evidence linking time perception to brain areas dedicated to motor control, namely the cerebellum, basal ganglia, premotor cortex, and supplementary motor area (SMA)^24–33^. Interestingly, the involvement of the motor system has also been demonstrated in tasks requiring the processing of auditory durations in the absence of motor timing^28,29,34,35^. The supplementary motor area, a region associated with cognitive aspects of motor control such as voluntary initiation/inhibition of an action, task switching, and learning action sequences^36^, is the brain area that is most consistently activated by time-related tasks, across modalities and temporal scales^26,27,37^. A BOLD topographical organization of duration selectivity (i.e., chronotopic maps) has been observed in the SMA during visual duration discrimination^38^, and functional connectivity measured during the same task has indicated this area as the final stage of duration processing^39^.

Despite this imaging evidence of the involvement of the motor system in duration perception, only one recent study has shown that adaptation to hand-tapping movements in mid-air can effectively bias perception of visual duration^9^. However, no studies have investigated the effects of motor duration adaptation (producing constant but not rhythmic durations) on the perceived duration of sensory stimuli.

In the present study, we induce adaptation through repeated motor production of a fixed duration and measure its aftereffect on auditory perception in a duration estimation task. Motor durations were produced through key presses that stimulated both tactile and proprioceptive systems. Given the impossibility of disentangling the individual contributions of these modalities, we refer to our procedure as “motor adaptation”.

We found that auditory adaptation affected auditory duration perception in agreement with previous findings^11^, while motor adaptation did not produce any aftereffect. These results suggest that auditory and motor durations are processed by independent mechanisms.

## Methods

### Ethical approval and recruitment of participants

The study was approved by the Medical Sciences Interdivisional Research Ethics Committee of the University of Oxford and complied with the Declaration of Helsinki. Sixteen volunteers (aged 18–40, mean age = 28.1 ± 7.3, 8 males) took part in this study. They gave written informed consent and were reimbursed at a rate of £15 per hour for participation, plus a bonus of up to £10 based on their performance. We conducted a power analysis based on the cross-modal (visual to auditory) aftereffect reported in Chen & He^18^: our sample size would allow us to detect an effect of similar size with 95% power.

### Stimuli and task

The task was programmed in MATLAB 2022 (The MathWorks Inc. (2022). MATLAB Version: 9.12.0.2039608 (R2022a) Update 5, Natick, Massachusetts: The MathWorks Inc. https://www.mathworks.com), using Psychtoolbox^40–42^ to generate all stimuli. Visual stimuli were displayed on a DELL E2417H monitor (60 Hz, 1920 × 1080 pixels, 23.8 inches) at a distance of about 70 cm. Auditory stimuli were presented through a pair of SONY MDR-ZX110 headphones.

The main task of duration estimation consisted of a single salient test tone, grossly above threshold, followed by a response reporting its duration. We used 1000-Hz tones with durations pseudo-randomly chosen between 200 ms and 2 s, in 41 logarithmic steps of 0.025 l.u., with each duration being tested 6 times in each condition. To avoid clicking noise artifacts, we added a 10-ms fade-in and fade-out, shaped as half of a Hanning window. The stimuli were superimposed on a continuously presented pink noise, whose intensity was set by the experimenter to effectively mask external noises in the room (including the participant’s key presses); the intensity of the tones was set to be ten times higher than that of the noise (equivalent to doubled perceived loudness), so their onset and offset would be clearly perceived. Participants estimated the tone duration by moving the mouse to adjust the length of a blue bar displayed vertically in the centre of the screen inside a black contour to represent a ‘meter’. After the tone offset, participants had 3 s to set its length as accurately as possible and confirm their response by pressing the left mouse button. The meter size was about 20° × 1°. Its full length corresponded to a duration of 2.2 s (allowing for a small margin in excess of the longest stimuli used). To establish the individual correspondence between temporal duration and visual length, participants practiced the task with feedback before the main sessions: as soon as the subject confirmed their response, the bar inside the meter switched from blue to red and showed the correct length, allowing them to calibrate the internal scale. The feedback lasted for 500 ms in each trial.

**Fig. 1:**
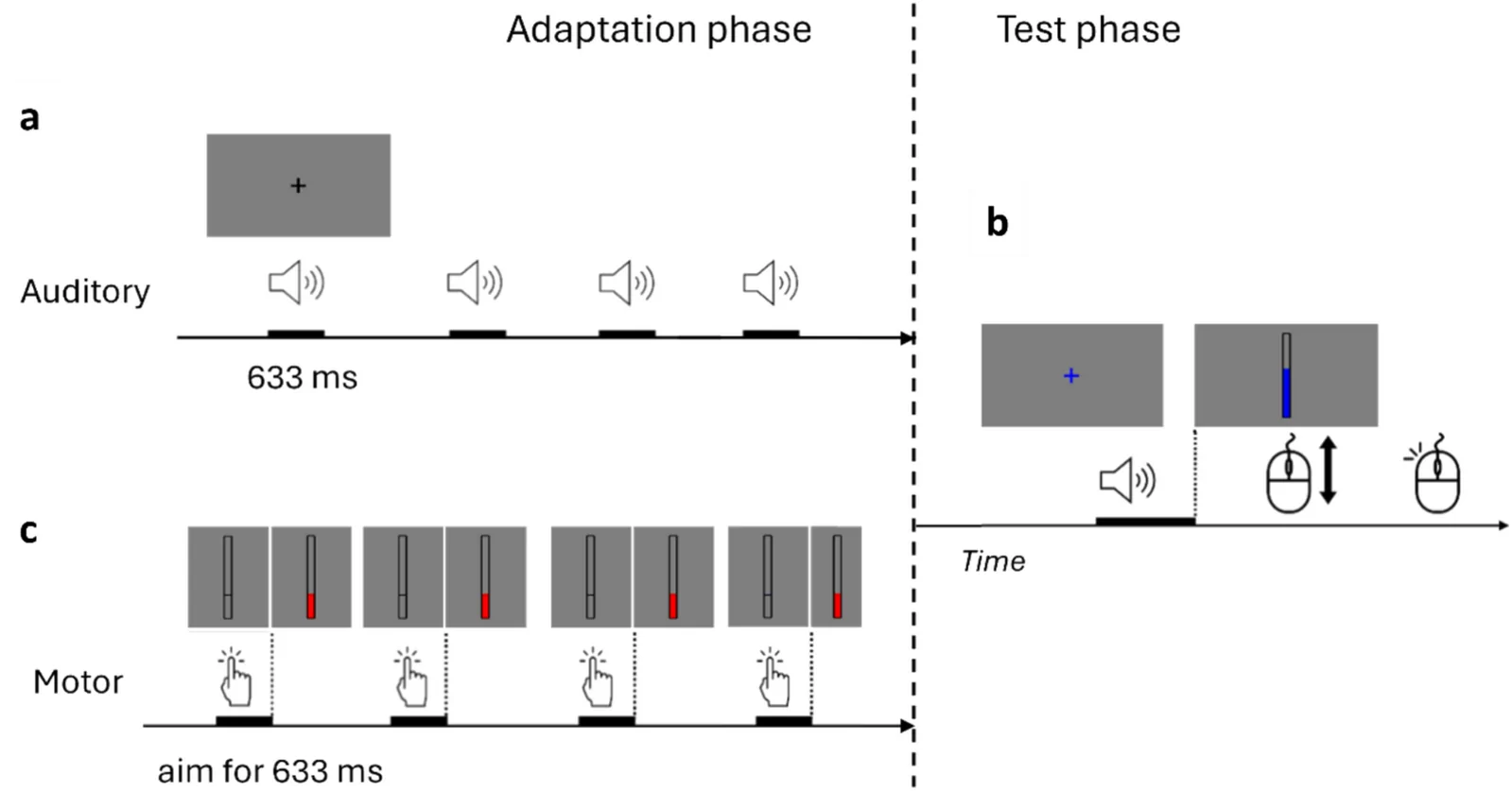
Trial structure for the adaptation conditions. (**a**) Auditory adaptation in the top-up phase of each trial with four adaptor tones. Black segments on the time axis represent the stimuli’s duration (constant at 633 ms). (**b**) Auditory duration estimation task. Participants hear a test tone and report its duration by adjusting the corresponding length (blue bar) on the meter. (**c**) Motor adaptation, similar to auditory adaptation, but each tone is replaced by a key press. The meter shows the target duration (black mark) and provides feedback (red bar) on the produced duration after the key is released. Dotted lines in (**b**) and (**c**) highlight simultaneity between the offset of auditory/motor events and the onset of visual events.

This task was performed in three conditions: baseline, auditory adaptation, and motor adaptation (Fig. 1).

#### Baseline condition

In the baseline condition, each trial started with the display of a black fixation cross (size: 1°) in the centre of the screen. After 3.2 s, the cross turned blue as a warning signal for the imminent test tone, whose onset was 250–1000 ms (randomly) after this transition. The response meter (blue bar in Fig. 1b) replaced the cross on the screen as soon as the tone was over, and it was displayed until the participant’s response or the 3-s timeout (after which, the message “Too slow… Press any key to continue” would be displayed).

#### Auditory adaptation condition

In the auditory adaptation condition (Fig. 1a), participants performed the estimation task again after adapting to an auditory duration of 633 ms, which corresponds to the geometric mean of all durations used in the test phase. The adaptation block started with a long series of 633-ms tones (100 for the first block, 50 for subsequent blocks). Then, 500 ms after the last tone’s offset, the message “Starting trials…” was displayed for 1 s. To maintain the adapting effect throughout the block, a 4-tone top-up was inserted before every trial. The inter-stimulus interval (ISI) for the adaptation tones, including the pause before the first, was randomized in the range 250–1000 ms. The fixation cross turned blue 200 ms after the fourth tone, followed by the test tone as in the baseline condition.

#### Motor adaptation condition

In the motor adaptation condition, the 633-ms tones used for auditory adaptation were replaced by key presses by the participant (Fig. 1c). The visual meter was used again to provide feedback, to help participants learn and keep a consistent pressing duration. The adaptation sequence began with the display of an empty meter, with a mark corresponding to the duration of 633 ms. Participants were required to press the ‘up’ arrow key for the equivalent duration. Only when they released the key, the produced duration was shown in red, so they could calibrate it with the mark and correct their error with the next key press. To replicate the random ISI of the auditory sequence, the feedback stayed on screen for a random amount of time (250–1000 ms, which also served to minimize any potential adapting effect of the visual stimuli), forcing participants to pause. Once the feedback disappeared and the meter was empty again, participants had only 1 s to start the next key press (or the message “Too slow… Press any key to continue” would be displayed, for a minimum of 500 ms). This ensured they maintained a pace roughly comparable to that of the auditory adaptation, while preventing them from engaging in rhythmic tapping. The only exception was the feedback to the fourth top-up key press in each trial, which had a fixed duration of 200 ms, right before the presentation of the fixation cross announcing the test. Participants used one hand to perform key presses for the motor adaptation, and the other hand to use the mouse to report auditory test durations.

#### Sessions

Participants completed 246 trials for each condition, in 6 blocks of 41 trials (with 1-min breaks). The study took place on two separate days (with 1 to 3 days in between). On each day, either the auditory or the motor adaptation condition was used (in counterbalanced order), while the baseline condition was split between both days. On each day, participants started by practicing the baseline task with feedback to learn the scale of the response meter (4 blocks of 41 trials), followed by 3 blocks of the baseline condition (without feedback). They then practiced the adaptation task (2 blocks of 21 and 20 trials), limiting the adaptation sequence to 10 and 5 events (instead of 100 and 50), to become familiar with the new task structure, without receiving any response feedback. After a 10-min break, added to allow any adapting effects to wear off, they re-practiced the baseline task with feedback (2 blocks of 41 trials), to consolidate their learning of the response scale, before concluding with the adaptation condition (6 blocks).

### Analysis

For each adaptation condition, the aftereffect was quantified as the difference in estimated durations with respect to the baseline. Statistical significance was established using a combination of nonlinear and linear models.

#### Nonlinear mixed-effects modelling

We used a nonlinear model to identify the dependence of auditory duration adaptation on the test duration. Applying a similar method as in Heron et al.^11^, we used the first derivative of a Gaussian to capture the repulsive nature of the aftereffect:

1 $$y = A \cdot x \cdot e^{{{\raise0.7ex\hbox{${ - x^{2} }$} \!\mathord{\left/ {\vphantom {{ - x^{2} } {2W^{2} }}}\right.\kern-0pt} \!\lower0.7ex\hbox{${2W^{2} }$}}}}$$

where *W* is the bandwidth (i.e., the standard deviation) and *A* is the amplitude. This function was adapted to the logarithmic scale of test durations (*D*) and centred on the adapted duration (*D*_*adapt*_) by substituting $\mathrm{x}=\mathrm{log}\left(\frac{D}{{D}_{adapt}}\right)$. Additionally, we multiplied this formula by a scaling function for reducing the effect amplitude at shorter durations and amplifying it at longer durations, an effect evident also in Heron et al.^11^. This is a feature we expected based on the well-known scalar properties of time perception^24,43^, and apparent in our data. We found that a square-root function of the test duration relative to the adapted duration ($\sqrt{\frac{D}{{D}_{adapt}}}$) resulted in a good fit of the data. Thus, the function that describes the changes of perceived duration after adaptation is:

2 $$A\sqrt{\frac{D}{{D}_{adapt}}}\bullet \mathrm{log}\left(\frac{D}{{D}_{adapt}}\right)\bullet {e}^{\frac{{-\mathrm{log}\left(\frac{D}{{D}_{adapt}}\right)}^{2}}{{2W}^{2}}}$$

Because this scaling function is not commonly used in modelling the adaptation effect, we also conducted the analysis excluding this term from the model: while the goodness of fit was reduced, the results confirmed our main findings (see supplementary material).

Lastly, we added a constant term (*C*) as both a fixed and a random effect, modelling a variable baseline offset across participants. This would reflect possible variation in response criterion between the baseline and the adaptation conditions, which were run in separate sessions (and days). We decided to include this term by comparing the fitting performance of the zero-constant model with that of the random-constant model; at the same time, we evaluated the inclusion of random effects for the parameters *A* and *W*. The best performing model, based on the lowest Bayesian Information Criterion, included random effects for the constant and for the amplitude, but fixed effect for the bandwidth:

3 $$Aftereffect = A\sqrt{\frac{D}{{D}_{adapt}}}\bullet \mathrm{log}\left(\frac{D}{{D}_{adapt}}\right)\bullet {e}^{\frac{{-\mathrm{log}\left(\frac{D}{{D}_{adapt}}\right)}^{2}}{{2W}^{2}}}+C$$

#### Linear modelling

To test the statistical significance of the adaptation effects, we restricted the analysis to the interval between the maximum and the minimum adaptation effect, where the data are well approximated by a linear function. We derived this interval from the non-linear model described above, and we applied a linear mixed-effects model to the difference in duration estimation between the adaptation condition and the baseline, with duration as the only fixed effect. In this analysis, the adaptation effect would manifest as a positive slope. We used the maximal model including random intercepts and random slopes for each individual participant’s data, after establishing that any reduced model resulted in a significantly lower fitting performance (according to a likelihood ratio test):

4 $$Aftereffect = 1+\mathrm{D}\mathrm{u}\mathrm{r}\mathrm{a}\mathrm{t}\mathrm{i}\mathrm{o}\mathrm{n}+\left( 1+\mathrm{D}\mathrm{u}\mathrm{r}\mathrm{a}\mathrm{t}\mathrm{i}\mathrm{o}\mathrm{n} \right| \mathrm{S}\mathrm{u}\mathrm{b}\mathrm{j}\mathrm{e}\mathrm{c}\mathrm{t})$$

To assess the variability across subjects, we also estimated the adaptation effect by applying a simple linear model to the data of individual participants (within the same range as above) in the baseline and adaptation conditions independently. We tested the presence of the adaptation effect (i.e., steeper slope) by applying a paired-sample *t-test* to the estimated slopes between each adaptation condition and the baseline. This test was complemented by the calculation of the Bayes Factor (for the alternative hypothesis that the adapted slope differs from the baseline slope), using the Bayes Factor package in R (R Core Team (2024). _R: A Language and Environment for Statistical Computing_. R Foundation for Statistical Computing, Vienna, Austria. https://www.R-project.org/; Morey R, Rouder J (2024). _BayesFactor: Computation of Bayes Factors for Common Designs_. R package version 0.9.12–4.7, https://CRAN.R-project.org/package=BayesFactor).

## Results

### Effect of auditory adaptation

**Fig. 2:**
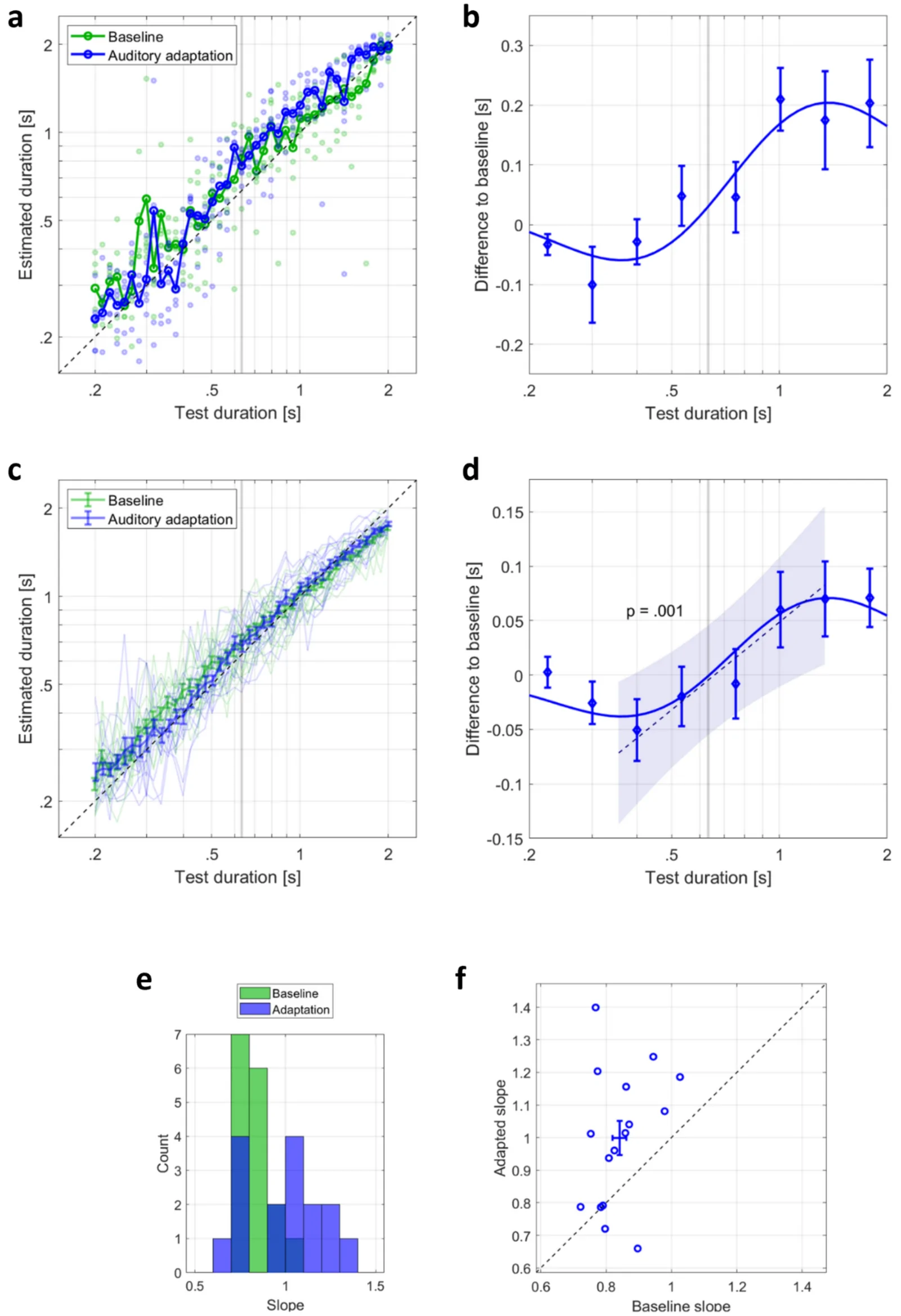
Effect of auditory adaptation. (**a**) Estimated durations in the baseline (green symbols) and auditory adaptation (blue symbols) conditions from one exemplar participant. Each light colour dot shows a single trial, dark colour dots and lines plot the average for each test duration. (**b**) Difference in estimated durations between conditions (adaptation—baseline), from the data in (**a**), averaged within equally spaced log interval bins. Bars are s.e.m. The curve is the result of the nonlinear model applied to this participant (see Eq. 3 in the Methods). (**c**) Average estimated durations of each participant (thin lines) and their average for each condition, colour code as in (**a**). Error bars are s.e.m across subjects. (**d**) Average difference in duration estimation between adaptation and baseline conditions (data are binned as in b). Error bars are s.e.m across subjects. The curve is the result of the nonlinear mixed-effects model. The dashed line is the prediction of the linear mixed-effects model, with the associated standard error. (**e**) Histogram of the slopes obtained from the linear fit of each participant’s estimated durations (**c**) within the test duration range highlighted in (**d**). Dark blue shows the overlap between baseline (green) and adaptation (light blue) conditions. (**f**) Scatter plot of the same data as in (**e**), showing individual participants (open circles) and their average with standard errors (cross). A paired-sample t-test revealed that the slopes differ significantly between the two conditions (t(15) = 3.10, p = .007, d = 0.78, Bayes Factor = 7.10).

Participants estimated a range of auditory durations, with or without prior auditory adaptation to 633 ms. Figure 2a and c show the estimated durations in both conditions for an example participant (a) and for the average of all participants (c). In the baseline condition, the duration estimates are not veridical, following instead a well-known compressive function resulting from central tendency bias^44–48^. In the adaptation condition, the duration estimates increased for stimuli longer than the adapted duration (633 ms), and decreased for stimuli shorter than the adapter. This produced functions of different slopes that crossed around the adapted duration. The differences in duration estimation between the adaptation condition and the baseline, calculated in equally spaced logarithmic bins of 0.125 l.u., produced a non-monotonic function (Fig. 2b and d). We estimated the difference data using the nonlinear mixed-effects model corresponding to the repulsive aftereffect expected from previous studies^11^ (Eq. 3). The result (blue curve in Fig. 2b and d) evaluated the position of the minimum and maximum at 362 ms (with an error evaluated by propagation of about 3%) and 1371 ms (with an error of about 6%) respectively, while modelling the data with a conditional R^2^ of 0.48 (see statistics in Table 1, see also supplementary Table S1).

**Table 1:** Results of nonlinear mixed-effects model analysis of auditory adaptation data. *A* amplitude, *W* width, *C* constant, τ variance of random effects, ρ covariance of random effects, σ^2^ error variance.

Fixed effects		
	Estimate	Std. Error
*A*	0.13	0.04
*W*	0.66	0.05
*C*	0.00	0.02

Random effects	
τ_A_	0.03
τ_C_	0.01
ρ_A–C_	0.00
N Subject	16
σ^2^	0.01
Marginal R^2^	0.078
Conditional R^2^	0.479
N observations	656

A linear mixed-effects model applied to the same data in the range between the minimum and the maximum of the function in Fig. 2d estimated a significantly positive slope (Table 2, Fig. 2d, see also Table S2, Figure S1a), indicating that the adaptation was effective in producing a repulsive bias in perceived duration. This conclusion was also supported by the individual-level linear modelling of estimated durations. The two slope distributions from the baseline vs adaptation conditions are shown in Fig. 2e and f (see also Figure S1b and c): the data from both conditions were approximately normally distributed (Shapiro–Wilk test, p = 0.30, and p = 0.83 respectively), with on average higher slopes in the adaptation condition (M = 1.00, SD = 0.21) vs baseline (M = 0.84, SD = 0.09). This result was statistically confirmed by a paired-sample t-test (t(15) = 3.10, p = 0.007, d = 0.78), and the associated Bayes Factor of 7.10.

**Table 2:** Results of linear mixed-effects model analysis of auditory adaptation data. τ variance of random effects, *ρ* covariance of random effects, σ^2^ error variance. Significant effects are in bold.

Fixed effects				
	Estimate	Std. Error	t-statistic	p-value
(Intercept)	0.05	0.03	1.61	0.108
Duration	0.12	0.04	3.22	**0.001**

Random effects	
τ_Int_	0.01
τ_Dur_	0.02
ρ_Int_-_Dur_	0.01
N Subject	16
σ^2^	0.01
Ordinary R^2^	0.556
Adjusted R^2^	0.555
Conditional R^2^	0.744
N observations	384

### Effect of motor adaptation

#### Motor production

We induced motor duration adaptation by having participants press a key repeatedly for 633 ms, with jittered intervals in between, to obtain a sequence whose timing would be as similar as possible to that of the auditory adapting stimuli.

**Fig. 3:**
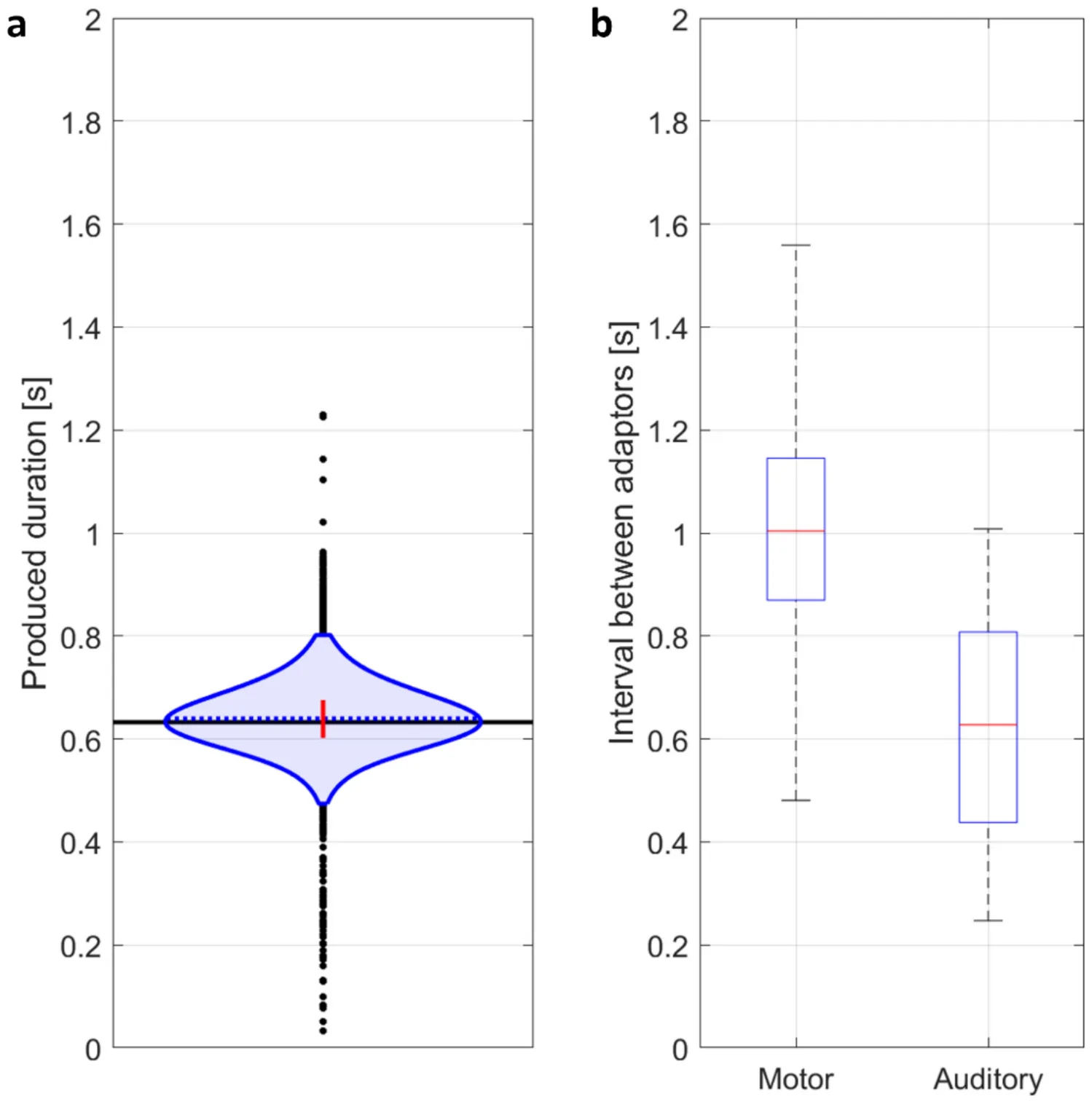
Motor production of adapting durations. (**a**) Violin plots showing the aggregate distribution of produced durations, aiming to produce the 663 ms duration (black line). The red line represents the interquartile range. Black dots are outliers (corresponding trials were excluded from analysis). (**b**) Box plots of intervals between produced durations and ISI in the auditory adaptation condition.

Figure 3a shows the distribution of produced durations aggregated across subjects. The average duration was 640 ms, with an inter-quartile range of 73 ms. Because adaptation would ideally require constant durations, we analysed the data both including all trials, and after excluding outliers: a trial was excluded if any of the four durations produced during the adaptation top-up was more than 3 median absolute deviation from the median of the aggregate distribution. This criterion resulted in boundaries of 474 and 802 ms, equivalent to a production error of about 25% of the 633 ms target duration. On average, 18.3 ± 22.4 trials (over 246) were excluded per participant, corresponding to ~ 7% of total trials. Here we report the results obtained after this selection; the analysis on the full dataset produced nearly identical outcomes.

Figure 3b shows the distribution of intervals between successive key presses aggregated across subjects, and the ISI during the auditory adaptation sequences. The mean motor interval averaged 1008 ms, longer than the mean auditory ISI of 626 ms, as expected from the task design. The motor inter-press variability was slightly lower, with a standard deviation of 189 ms vs 216 ms for auditory adaptation. We comment on these differences in the discussion.

#### Lack of adaptation

**Fig. 4:**
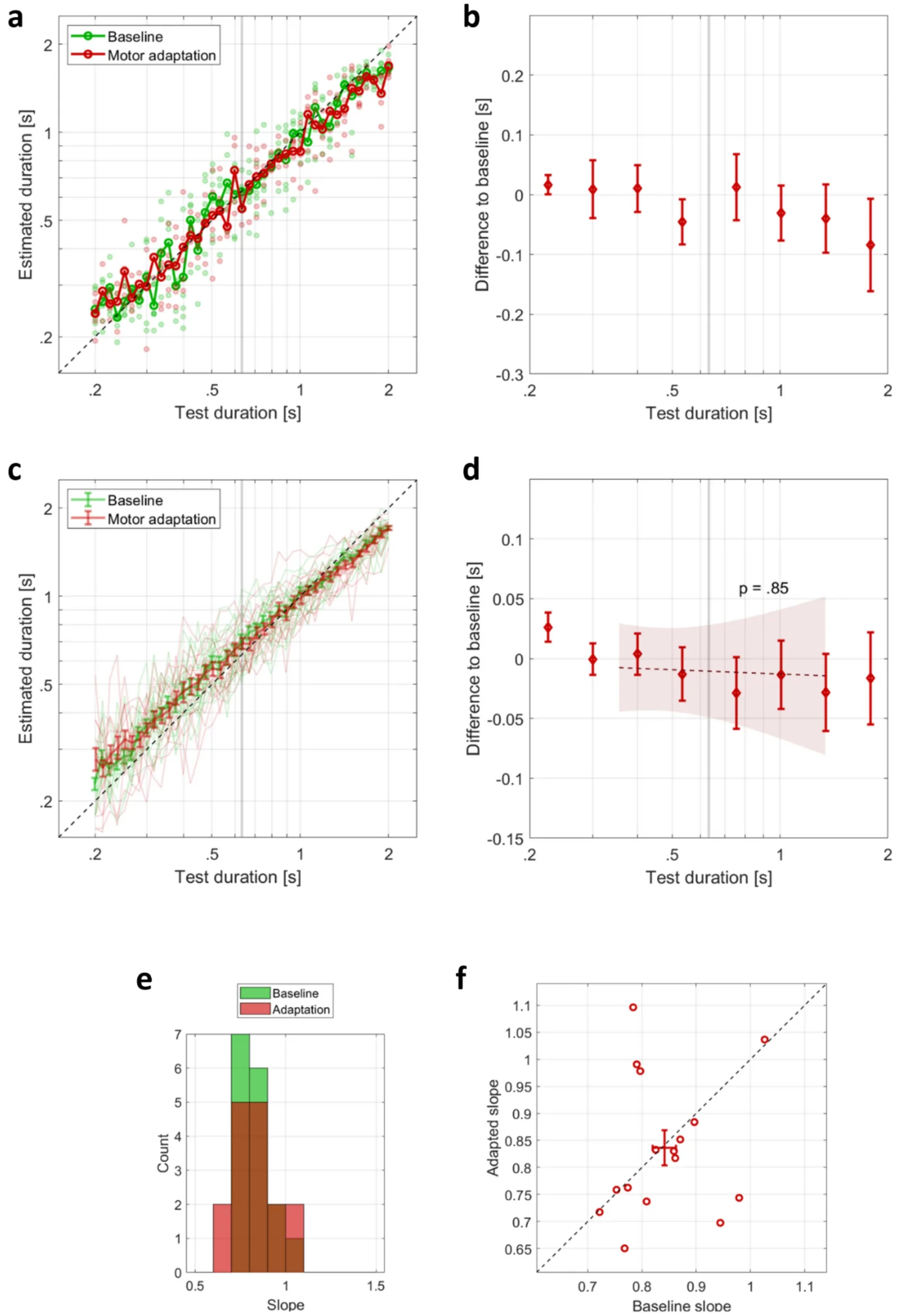
Effect of motor adaptation. (**a**) Estimated durations in the baseline (green symbols) and motor adaptation (red symbols) conditions from one exemplar participant. Each light colour dot shows a single trial, dark colour dots and lines plot the average for each test duration. (**b**) Difference in estimated durations between conditions (adaptation—baseline), from the data in (**a**), averaged within equally spaced log interval bins. Bars are s.e.m. (**c**) Average estimated durations of each participant (thin lines) and their average for each condition, colour code as in (**a**). Error bars are s.e.m across subjects. (**d**) Average difference in duration estimation between adaptation and baseline conditions (data are binned as in b). Error bars are s.e.m across subjects. The dashed line is the prediction of the linear mixed-effects model, with the associated standard error. (**e**) Histogram of the slopes obtained from linear fitting of each participant’s estimated durations within the range highlighted by the linear model in (**d**). Dark red shows the overlap between baseline (green) and adaptation (light red) conditions. (**f**) Scatter plot of the same data as in (**e**), showing individual participants and their average with standard errors. No significant difference was found between conditions (Bayes Factor = 0.26).

Figure 4a and c show the estimated durations in the motor adaptation and baseline conditions for an example participant (a) and for the average across all participants (c). Here, the baseline and the adaptation curves overlap, suggesting that motor adaptation does not affect auditory duration perception. The binned difference curves (Fig. 4b and d, see also Figure S1d) are practically constant over the range of tested durations. We assessed the adaptation effect using the same linear model analysis as for the auditory condition, and applying it to the same range of test durations (356 to 1337 ms). Panel D also shows the result of the linear mixed model, showing no trend in the difference-to-baseline data (statistics are reported in Table 3, see also Table S3).

**Table 3:** Statistical results of linear mixed-effects modelling of motor adaptation data. τ variance of random effects, ρ covariance of random effects, σ^2^ error variance.

Fixed effects				
	Estimate	Std. Error	t-statistic	p-value
(Intercept)	− 0.01	0.03	− 0.47	0.637
Duration	− 0.01	0.03	− 0.19	0.846

Random effects	
τ_Int_	0.01
τ_Dur_	0.01
ρ_Int_-_Dur_	0.01
N Subject	16
σ^2^	0.01
Ordinary R^2^	0.360
Adjusted R^2^	0.359
Conditional R^2^	0.610
N observations	384

The individual-level linear modelling of estimated durations leads to an equivalent outcome. The distribution of slopes from the motor adaptation condition was approximately normal (Shapiro–Wilk test, p = 0.33), and very similar to that from the baseline condition (M = 0.84, SD = 0.13 vs M = 0.84, SD = 0.09 for the baseline, see Fig. 4e and f, see also Figure S1e and f). Expectedly, the paired-sample t-test also failed to detect a difference (t(15) = − 0.13, p = 0.90, d = − 0.03), while the Bayes Factor was estimated to be 0.26, equivalent to a factor of 3.86 in favour of the absence of an effect of motor adaptation.

**Fig. 5:**
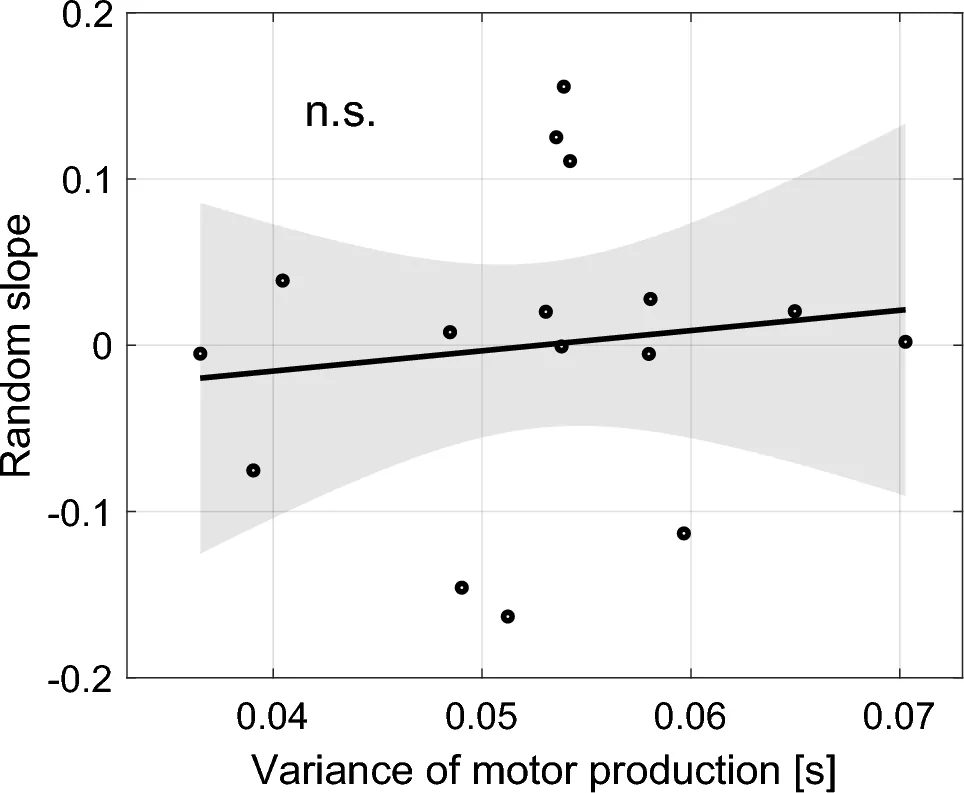
Absence of correlation between the precision of motor adaptation (inversely quantified as the variance of produced durations, x-axis) and the strength of the duration aftereffect (quantified as the random slope of the duration effect from the nonlinear model, y-axis) across subjects.

Finally, we correlated the random slopes obtained from the linear mixed model with the variance of each participant’s motor production during adaptation, to assess whether the participants’ precision in producing the adaptor duration could at least partially explain the absence of the duration aftereffect. The variance of motor production varied by approximately a factor of 2 across participants (from 37 to 70 ms), but it did not explain variability in the adaptation effect (R^2^ = 0.01; p = 0.65, Fig. 5). The associated Bayes Factor of 0.56 also supports a lack of correlation, indicating that the null result from the main analysis was not substantially determined by the participants’ ability (or lack thereof) to produce constant durations.

## Discussion

The present study is the first to explore the effects of adaptation to motor duration on the perception of auditory duration, despite the strong evidence discussed in the introduction of the involvement of the motor system in time duration processing. We used a duration estimation task and observed an aftereffect only following auditory adaptation.

The fact that auditory duration perception was not affected by motor adaptation suggests that duration processing is independent between the auditory and the motor modalities. This may seem to contrast with the ubiquitous involvement of the motor system in timing tasks. Imaging studies have pointed to the basal ganglia and SMA as the core network for temporal processing^29–31,33,37,41,56–65^. Coull et al.^49^, in particular, used high-temporal resolution fMRI to measure brain activity at different stages during a visual duration discrimination task, finding that the SMA is the only area activated by both the reference and comparison stimuli (consistent with a timing function), whereas the putamen is mostly activated by the first stimulus, suggesting a role in the storage of temporal information rather than timing per se. Moreover, BOLD responses in the SMA are organized chronotopically^38^, and their amplitude correlates with subjective duration^50^, strongly indicating a functional specialization for temporal processing.

Motor signals also modulate the perception of sensory time. A well-known phenomenon in which motor signals influence visual duration is chronostasis, the expansion of time following a voluntary action (saccades, hand movements, or vocal utterances)^51^. Duration expansion has also been observed during the preparation of hand movements^52^. Similarly, the perception of empty intervals can be compressed or expanded by the execution of eye or hand movements^53–55^ . The timing of the consequence of one’s action^56^ or the timing of go-cues for an action^57^ is attracted toward the time of action execution, demonstrating similar time distortions in the sensory and motor systems.

The absence of a transfer of motor duration adaptation to sensory duration channels may not be in contrast with the above evidence. Firstly, no evidence is available on how all those effects change after adaptation of the motor duration mechanism, and secondly, different clocks may operate simultaneously in the motor system. In our task, subjects were allocating attention to start and stop the key press at the appropriate time, and this presumably required a precise programming of the movement. It is plausible that the clock associated with the intention-to-move signals is more strongly affected by our motor adaptation. However, whatever might be the origin of the motor clock that adapts in our experiment, we should stress that motor timing must have different properties from sensory timing mechanisms. Motor processes monitor temporal duration predictively or while the action is elapsing, most likely by means of some kind of accumulator which computes the appropriate time to trigger an action (such as the release of the key press in our task) in advance. On the contrary, the perception of duration of sensory input may rely on mechanisms that compute the duration of a stimulus only after it has elapsed. It is possible that the duration we store in short-term memory right after stimulus offset derives from timing mechanisms that function postdictively and hence cannot be modulated by the predictive motor clock.

Postdiction is the phenomenon by which our perception of a stimulus also depends on sensory information available only after it has elapsed^58–61^. This process has been shown to play a role in several illusions^62^ and to act even cross-modally^63^, suggesting that it is a general principle of the way the brain interprets the external world: by collecting information over a temporal window that includes the immediate future of a stimulus, and processing it through operations similar to temporal smoothing^64^, the brain would achieve a more reliable (although delayed) perception. Notably, postdiction is believed to play a role in determining how sensory stimuli are consciously perceived, but not in the way the same stimuli may unconsciously affect motor control, since allowing such a processing delay would impair our ability to quickly react to external events^58,59^. This distinction between real-time sensory processing for action and postdictive sensory processing for perception may explain the lack of motor adaptation on sensory duration mechanisms demonstrated here. According to this interpretation, the commonly observed activation of motor-related brain structures during perceptual timing tasks would reflect shared mechanisms (between action and perception) for online temporal processing, while the repulsive duration aftereffect would reflect adaptation of a postdictive timing system exclusive to sensory processing.

While the focus of the present study was the motor modality, we should consider that each key press of the motor adaptation sequence also provided a tactile stimulus, therefore our results also show that auditory duration perception was not biased by tactile duration adaptation. This question has been previously explored by Li et al.^65^, who also found no evidence for a cross-modal aftereffect between touch and audition (reciprocally). However, they used adaptors of the same physical duration in the two modalities, instead of matching them in perceived duration, which has recently been shown to be crucial for this type of experiment^18^. The design we used, instead, would allow us to detect a cross-modal aftereffect regardless of cross-modal differences in perceived duration by testing a wide range of durations around the adaptor: in this way, differences in subjective duration between the adapted and test modalities would manifest in Fig. 4b as a horizontal shift of the repulsive modulation while its profile would remain unaltered. Contrary to this scenario, our data clearly show the absence of any adaptation effect.

The lack of interaction between touch and audition may seem at odds with the disruptive effect of TMS on tactile duration perception when applied to the auditory cortex^66^. One possible explanation may be related to TMS signals spreading from the application site to connected brain areas^67–69^, so the apparent cross-modal effect may reflect a limited specificity of this technique. Another possibility is that auditory and tactile duration mechanisms are partially shared, but adaptation does not occur for tactile stimuli that are produced by intentional actions. Tactile adaptation has been shown to be completely reset after a voluntary action^70^, showing that there is a strong cross-link between tactile duration adaptation and intention-to-move signals. This interpretation corroborates the idea that the adaptation in our experiment involves mainly the intention-to-move signals, as discussed above.

### Motor duration vs tapping adaptation

Contrary to our results, Anobile et al.^9^ found a cross-modal motor adaptation effect on subjective duration, showing that adaptation to hand-tapping movements in mid-air alters the perceived visual duration of drifting gratings. They asked participants to tap at a rate of about once per second vs “as fast as possible” and then assessed their perception of a 600-ms target duration, demonstrating that slow tapping caused duration expansion while fast tapping caused duration compression. Similar to the adaptation to temporal frequency-modulated stimuli (see Introduction), tapping adaptation simultaneously biases the perception of spatial and temporal numerosity^9,71^ of visual stimuli, with effects that are localized around the tapping hand. Time mechanisms are modulated by the amount of information flow. In Anobile et al.^9^, as well as in Johnston et al.^7^ and Burr et al.^6^, the adapter is a continuous flow of events that can in principle modulate the clocks, as it is well known that a high-rate flow of information induces time expansion^72^. The interaction between the rate of the movement and the rate of the visual drift of target stimuli may explain their results: the neuronal combination of the two information rates may be generating the duration after-effect. This explanation would be consistent with the simultaneous effect they found on numerosity and duration^9,10^.

The experiment of Anobile et al.^9^ does not address the existence or selectivity of the visual duration channels, which constitutes a major goal of the present study. Rather than hand tapping, we adapted the motor execution of a precisely timed duration of 633 ms. The two types of motor adaptation might not engage the same timing mechanisms: the degree of intentional control these actions require is clearly different. In our task, participants needed to attend to each key press as an independent event, whereas repeated tapping may be implemented through more automatic mechanisms relying on the processing of rhythmic patterns.

### Limitations given by the motor adaptation design

An obvious concern raised by our results is whether the motor adaptation procedure we used is suitable for adapting duration processing in the dedicated clock in a measurable way. Motor adaptation relies on the participant’s ability to produce movements according to the study’s requirements, unlike sensory adaptation, which depends on stimuli precisely controlled by the experimenter. This introduces differences between the sensory and the motor conditions that go beyond the simple change in modality.

Duration adaptation would require, ideally, the repetition of a fixed duration, but the inevitable variability in motor duration production around the target duration might cause the adapting effect to spread over a larger range of duration channels, compared to what is achievable with sensory stimulation. If this spread caused a substantial weakening of the aftereffect, we would observe a correlation between the precision of motor production and the strength of the effect, contrary to what we found. Therefore, the larger variability in the motor (vs auditory) adaptation is unlikely to explain the lack of an aftereffect. On the other hand, the inter-press interval was intended to be randomized to avoid introducing rhythmic patterns in the adapting sequence, as these might engage different timing mechanisms from those processing single durations^25,73–75^. To this goal, since most participants have a spontaneous tendency to press the response key rhythmically, the timing of their motor production was dictated by a cue (the feedback’s offset), which successfully made the adapting sequence non-rhythmic, with a similar variability to that of the auditory ISI (Fig. 3b). Although the participants’ reaction times made the average inter-press interval longer than the average ISI, we deem this difference negligible, given previous studies suggesting that the length of the ISI does not substantially interfere with duration adaptation^11,12^.

Another difference between the auditory and the motor adaptation procedures lies in the visual presentation of the production feedback. While it is evident from the results that the duration of visual stimuli is not affecting auditory estimation performance, a potential concern might arise in relation to visual size adaptation^76^. The length shown on the meter after each production was approximately constant (as constant as the produced durations), and its repeated presentation might have caused a distortion of perceived length that would bias the estimation responses in such a way as to compensate for the perceptual bias induced by motor adaptation. In this regard, it should be noted that, when reporting each estimate, the length of the response bar was adjusted by participants inside the meter’s empty contour, which constituted a reference. This would have prevented an influence of visual size adaptation, as this phenomenon would not alter the participants’ perception of the *proportion* between the length of the bar and that of the meter.

Another possible concern raised by the presentation of the feedback bar after each key press is the possibility that participants learned a motor-sensory association between their actions and the visual length representing the adapted duration. Learned associations can affect behavioural responses in some tasks^77^. This effect, however, only occurs if those responses involve sensory or motor features (such as the motor effector and spatial location involved) that overlap substantially with those of the learned event^77^. We took particular care to mitigate this overlap. First, participants used the keyboard and the mouse with different hands (and obviously, the physical location of each device was also different). Second, the action was a temporally controlled key press on a keyboard vs a spatially controlled mouse movement and click. Third, the bar was shown after each adaptation key press, whereas task responses were given while adjusting the bar length in real time. Given these differences in the actions being performed during adaptation and task-response phases, an interference of action–effect bindings during the adaptation phase on the task response is very unlikely.

Finally, participants might have timed their key presses based on the duration of the tactile feedback, while ignoring the duration of their motor command, thus minimizing motor adaptation. We find such a strategy very unlikely, since it would imply dismissing a useful source of temporal information, contrary to the goal of optimizing timing performance. Instead, we believe that tactile duration would be integrated with motor duration, in which case the latter would still play a major role in the timing process, as intended by the task design. Similar considerations apply to proprioceptive feedback: considering that motor and proprioceptive functions are systematically associated, we might expect duration processing in the respective modalities to be tightly coupled. It is known that the duration of movements can be integrated with the duration of sensory stimuli, and their Bayesian combination leads to an improvement in duration perception^78–80^.

### Conclusions

We have replicated the effect of duration adaptation within the auditory modality as found by previous studies^11^, using an estimation task that allows us to isolate the aftereffect in the test modality from any concurrent effects in other modalities. Using the same estimation method, we did not observe any effect of motor adaptation on auditory duration perception. This finding suggests that motor duration processing, probably of predictive nature, is an independent mechanism from the one that determines our perception of auditory duration.

## Supplementary Information

Supplementary Information.

## Data Availability

The datasets generated during and/or analysed during the current study are available from the corresponding author on reasonable request.

## References

[1] Wearden, J. H. & Lejeune, H. Scalar properties in human timing: Conformity and violations. *Quart. J. Exp. Psychol.* **61**, 569–587 (2008). 10.1080/17470210701282576 18938276

[2] Rammsayer, T. H. Differences in duration discrimination of filled and empty auditory intervals as a function of base duration. *Atten. Percept. Psychophys.* **72**, 1591–1600 (2010). 20675803 10.3758/APP.72.6.1591

[3] Rammsayer, T. H. The effects of type of interval, sensory modality, base duration, and psychophysical task on the discrimination of brief time intervals. *Atten. Percept. Psychophys.* **76**, 1185–1196 (2014). 24596081 10.3758/s13414-014-0655-x

[4] Westheimer, G. Discrimination of short time intervals by the human observer. *Exp. Brain Res.* **129**, 121–126 (1999). 10550509 10.1007/s002210050942

[5] Rammsayer, T. H. & Lima, S. D. Duration discrimination of filled and empty auditory intervals: Cognitive and perceptual factors. *Percept. Psychophys.* **50**, 565–574 (1991). 1780204 10.3758/bf03207541

[6] Burr, D. C., Cicchini, G. M., Arrighi, R. & Morrone, M. C. Spatiotopic selectivity of adaptation-based compression of event duration. *J. Vis.* **11**, 21–21 (2011). 21357369 10.1167/11.2.21

[7] Johnston, A., Arnold, D. H. & Nishida, S. Spatially localized distortions of event time. *Curr. Biol.* **16**, 472–479 (2006). 16527741 10.1016/j.cub.2006.01.032

[8] Burr, D., Tozzi, A. & Morrone, C. Neural mechanisms for timing visual events are spatially selective in real-world coordinates. *Nat. Neurosci.* **10**, 423–425 (2007). 17369824 10.1038/nn1874

[9] Anobile, G., Domenici, N., Togoli, I., Burr, D. & Arrighi, R. Distortions of visual time induced by motor adaptation. *J. Exp. Psychol. Gen.* **149**, 1333–1343 (2020). 31789572 10.1037/xge0000709

[10] Burr, D. C., Ross, J., Binda, P. & Morrone, M. C. Saccades compress space, time and number. *Trends Cogn. Sci.* **14**, 528–533 (2010). 20971675 10.1016/j.tics.2010.09.005

[11] Heron, J. et al. Duration channels mediate human time perception. *Proc. R. Soc. B: Biol. Sci.* **279**, 690–698 (2012). 10.1098/rspb.2011.1131 PMC3248727 21831897

[12] Li, B., Yuan, X. & Huang, X. The aftereffect of perceived duration is contingent on auditory frequency but not visual orientation. *Sci. Rep.* **5**, 10124 (2015). 26054927 10.1038/srep10124 PMC4460570

[13] Heron, J., Hotchkiss, J., Aaen-Stockdale, C., Roach, N. W. & Whitaker, D. A neural hierarchy for illusions of time: Duration adaptation precedes multisensory integration. *J. Vis.* **13**, 1–12 (2013). 10.1167/13.14.4 PMC3852255 24306853

[14] Maarseveen, J., Paffen, C. L. E., Verstraten, F. A. J. & Hogendoorn, H. The duration aftereffect does not reflect adaptation to perceived duration. *PLoS ONE* **14**, e0213163 (2019). 30830930 10.1371/journal.pone.0213163 PMC6398839

[15] Heron, J., Fulcher, C., Collins, H., Whitaker, D. & Roach, N. W. Adaptation reveals multi-stage coding of visual duration. *Sci. Rep.* **9**, 3016 (2019). 30816131 10.1038/s41598-018-37614-3 PMC6395619

[16] Walker, J. T., Irion, A. L. & Gordon, D. G. Simple and contingent aftereffects of perceived duration in vision and audition. *Percept. Psychophys.* **29**, 475–486 (1981). 7279574 10.3758/bf03207361

[17] Walker, J. T. & Irion, A. L. Two new contingent aftereffects: Perceived auditory duration contingent on pitch and on temporal order. *Percept. Psychophys.* **26**, 241–244 (1979). 537859 10.3758/bf03199875

[18] Chen, H. & He, S. Duration adaptation depends on the perceived rather than physical duration and can be observed across sensory modalities. *Perception* **54**, 180–195 (2025). 39871719 10.1177/03010066251314184

[19] Rohde, M. & Ernst, M. O. Time, agency, and sensory feedback delays during action. *Curr. Opin. Behav. Sci.* **8**, 193–199 (2016).

[20] Sugano, Y., Keetels, M. & Vroomen, J. Adaptation to motor-visual and motor-auditory temporal lags transfer across modalities. *Exp. Brain Res.* **201**, 393–399 (2010). 19851760 10.1007/s00221-009-2047-3 PMC2832876

[21] Sugano, Y., Keetels, M. & Vroomen, J. Audio-motor but not visuo-motor temporal recalibration speeds up sensory processing. *PLoS ONE* **12**, e0189242 (2017). 29216307 10.1371/journal.pone.0189242 PMC5720774

[22] Stetson, C., Cui, X., Montague, P. R. & Eagleman, D. M. Motor-sensory recalibration leads to an illusory reversal of action and sensation. *Neuron* **51**, 651–659 (2006). 16950162 10.1016/j.neuron.2006.08.006

[23] Ayhan, I., Kurtcan, M. & Thorpe, L. The effect of action on perceptual feature binding. *Vision Res.* **177**, 97–108 (2020). 33007649 10.1016/j.visres.2020.09.001

[24] Buhusi, C. V. & Meck, W. H. What makes us tick? Functional and neural mechanisms of interval timing. *Nat. Rev. Neurosci.* **6**, 755–765 (2005). 16163383 10.1038/nrn1764

[25] Teki, S., Grube, M., Kumar, S. & Griffiths, T. D. Distinct neural substrates of duration-based and beat-based auditory timing. *J. Neurosci.* **31**, 3805–3812 (2011). 21389235 10.1523/JNEUROSCI.5561-10.2011 PMC3074096

[26] Wiener, M., Turkeltaub, P. & Coslett, H. B. The image of time: A voxel-wise meta-analysis. *Neuroimage* **49**, 1728–1740 (2010). 19800975 10.1016/j.neuroimage.2009.09.064

[27] Lewis, P. A. & Miall, R. C. Distinct systems for automatic and cognitively controlled time measurement: Evidence from neuroimaging. *Curr. Opin. Neurobiol.* **13**, 250–255 (2003). 12744981 10.1016/s0959-4388(03)00036-9

[28] Schubotz, R. I., Friederici, A. D. & Yves Von Cramon, D. Time perception and motor timing: A common cortical and subcortical basis revealed by fMRI. *Neuroimage* **11**, 1–12 (2000). 10686112 10.1006/nimg.1999.0514

[29] Shih, L. Y. L., Kuo, W. J., Yeh, T. C., Tzeng, O. J. L. & Hsieh, J. C. Common neural mechanisms for explicit timing in the sub-second range. *NeuroReport* **20**, 897–901 (2009). 19451837 10.1097/WNR.0b013e3283270b6e

[30] Schwartze, M., Rothermich, K. & Kotz, S. A. Functional dissociation of pre-SMA and SMA-proper in temporal processing. *Neuroimage* **60**, 290–298 (2012). 22178297 10.1016/j.neuroimage.2011.11.089

[31] Ivry, R. B., Spencer, R. M., Zelaznik, H. N. & Diedrichsen, J. The cerebellum and event timing. *Ann. N. Y. Acad. Sci.* **978**, 302–317 (2002). 12582062 10.1111/j.1749-6632.2002.tb07576.x

[32] Bareš, M. et al. Consensus paper: Decoding the contributions of the cerebellum as a time machine. From neurons to clinical applications. *The Cerebellum* **18**, 266–286 (2019). 30259343 10.1007/s12311-018-0979-5

[33] Matell, M. S. & Meck, W. H. Cortico-striatal circuits and interval timing: Coincidence detection of oscillatory processes. *Cogn. Brain Res.* **21**, 139–170 (2004). 10.1016/j.cogbrainres.2004.06.012 15464348

[34] Belin, P. et al. The neuroanatomical substrate of sound duration discrimination. *Neuropsychologia* **40**, 1956–1964 (2002). 12207993 10.1016/s0028-3932(02)00062-3

[35] Tregellas, J. R., Davalos, D. B. & Rojas, D. C. Effect of task difficulty on the functional anatomy of temporal processing. *Neuroimage* **32**, 307–315 (2006). 16624580 10.1016/j.neuroimage.2006.02.036

[36] Nachev, P., Kennard, C. & Husain, M. Functional role of the supplementary and pre-supplementary motor areas. *Nat. Rev. Neurosci.* **9**, 856–869 (2008). 18843271 10.1038/nrn2478

[37] Nani, A. et al. The neural correlates of time: A meta-analysis of neuroimaging studies. *J. Cogn. Neurosci.* **31**, 1796–1826 (2020). 10.1162/jocn_a_01459 31418337

[38] Protopapa, F. et al. Chronotopic maps in human supplementary motor area. *PLoS Biol.* **17**, e3000026 (2019). 30897088 10.1371/journal.pbio.3000026 PMC6428248

[39] Protopapa, F., Kulashekhar, S., Hayashi, M. J., Kanai, R. & Bueti, D. Effective connectivity in a duration selective cortico-cerebellar network. *Sci. Rep.* **13**, 20674 (2023). 38001253 10.1038/s41598-023-47954-4 PMC10673930

[40] Brainard, D. H. The psychophysics toolbox. *Spat. Vis.* 10.1163/156856897X00357 (1997). 9176952

[41] Pelli, D. G. The VideoToolbox software for visual psychophysics: Transforming numbers into movies. *Spat. Vis.* 10.1163/156856897X00366 (1997). 9176953

[42] Kleiner, M. et al. What’s new in Psychtoolbox-3?. *Perception*10.1068/v070821 (2007).

[43] Malapani, C. & Fairhurst, S. Scalar timing in animals and humans. *Learn. Motiv.* **33**, 156–176 (2002).

[44] Cicchini, G. M., Arrighi, R., Cecchetti, L., Giusti, M. & Burr, D. C. Optimal encoding of interval timing in expert percussionists. *J. Neurosci.* **32**, 1056–1060 (2012). 22262903 10.1523/JNEUROSCI.3411-11.2012 PMC6621155

[45] Zimmermann, E. & Cicchini, G. M. Temporal Context affects interval timing at the perceptual level. *Sci. Rep.* **10**, 8767 (2020). 32472083 10.1038/s41598-020-65609-6 PMC7260213

[46] Roach, N. W., McGraw, P. V., Whitaker, D. J. & Heron, J. Generalization of prior information for rapid Bayesian time estimation. *Proc. Natl. Acad. Sci. U. S. A.* **114**, 412–417 (2017). 28007982 10.1073/pnas.1610706114 PMC5240697

[47] Damsma, A., Schlichting, N. & van Rijn, H. Temporal context actively shapes EEG signatures of time perception. *J. Neurosci.* **41**, 4514–4523 (2021). 33833083 10.1523/JNEUROSCI.0628-20.2021 PMC8152605

[48] Hollingworth, H. L. The central tendency of judgment. *J. Philos. Psychol. Sci. Methods* **7**(17), 461–469 (1910).

[49] Coull, J. T., Nazarian, B. & Vidal, F. Timing, storage, and comparison of stimulus duration engage discrete anatomical components of a perceptual timing network. *J. Cogn. Neurosci.* **20**, 2185–2197 (2008). 18457512 10.1162/jocn.2008.20153

[50] Tipples, J., Brattan, V. & Johnston, P. Neural bases for individual differences in the subjective experience of short durations (Less than 2 Seconds). *PLoS ONE* **8**, e54669 (2013). 23342176 10.1371/journal.pone.0054669 PMC3547013

[51] Park, J., Schlag-Rey, M. & Schlag, J. Voluntary action expands perceived duration of its sensory consequence. *Exp. Brain Res.* **149**, 527–529 (2003). 12677334 10.1007/s00221-003-1376-x

[52] Hagura, N., Kanai, R., Orgs, G. & Haggard, P. Ready steady slow: action preparation slows the subjective passage of time. *Proc. R. Soc. B: Biol. Sci.* **279**, 4399–4406 (2012). 10.1098/rspb.2012.1339 PMC3479796 22951740

[53] Tomassini, A., Gori, M., Baud-Bovy, G., Sandini, G. & Morrone, M. C. Motor commands induce time compression for tactile stimuli. *J. Neurosci.* **34**, 9164–9172 (2014). 24990936 10.1523/JNEUROSCI.2782-13.2014 PMC4112941

[54] Binda, P., Cicchini, G. M., Burr, D. C. & Morrone, M. C. Spatiotemporal distortions of visual perception at the time of saccades. *J. Neurosci.* **29**(42), 13147–13157. 10.1523/JNEUROSCI.3723-09.2009 (2009). 19846702 PMC6665185

[55] Morrone, M. C., Ross, J. & Burr, D. Saccadic eye movements cause compression of time as well as space. *Nat. Neurosci.* **8**(7), 950–954. 10.1038/nn1488 (2005). 15965472

[56] Haggard, P., Clark, S. & Kalogeras, J. Voluntary action and conscious awareness. *Nat. Neurosci.* **5**, 382–385 (2002). 11896397 10.1038/nn827

[57] Yabe, Y. & Goodale, M. A. Time flies when we intend to act: Temporal distortion in a Go/No-Go task. *J. Neurosci.* **35**, 5023–5029 (2015). 25810531 10.1523/JNEUROSCI.4386-14.2015 PMC6705366

[58] Eagleman, D. M. How does the timing of neural signals map onto the timing of perception? In *Space and Time in Perception and Action* (eds Nijhawan, R. & Khurana, B.) (Cambridge University Press, 2010).

[59] Herzog, M. H., Drissi-Daoudi, L. & Doerig, A. All in good time: Long-lasting postdictive effects reveal discrete perception. *Trends Cogn. Sci.* **24**, 826–837 (2020). 32893140 10.1016/j.tics.2020.07.001

[60] Eagleman, D. M. & Sejnowski, T. J. Motion integration and postdiction in visual awareness. *Science* **1979**(287), 2036–2038 (2000). 10.1126/science.287.5460.2036 10720334

[61] Kolers, P. A. & Grunau, M. Von. Shape and color in apparent motion. *Vision Res* **16**, 329–335 (1976). 10.1016/0042-6989(76)90192-9 941407

[62] Shimojo, S. & Yamada, Y. Postdiction: Its implications on visual awareness, hindsight, and sense of agency. *Front. Psychol.* **5**, 196 (2014). 24744739 10.3389/fpsyg.2014.00196 PMC3978293

[63] Stiles, N. R. B., Tanguay, A. R. & Shimojo, S. Crossmodal postdiction: Conscious perception as revisionist history. *J. Perceptual Imaging* **5**, jpi0150 (2022). 10.2352/J.Percept.Imaging.2022.5.000403 PMC9028020 35464341

[64] Rao, R. P. N., Eagleman, D. M. & Sejnowski, T. J. Optimal smoothing in visual motion perception. *Neural Comput.* **13**, 1243–1253 (2001). 11387045 10.1162/08997660152002843

[65] Li, B., Chen, L. & Fang, F. Somatotopic representation of tactile duration: evidence from tactile duration aftereffect. *Behav. Brain Res.* **371**, 111954 (2019). 31121218 10.1016/j.bbr.2019.111954

[66] Bolognini, N., Papagno, C., Moroni, D. & Maravita, A. Tactile temporal processing in the auditory cortex. *J. Cogn. Neurosci.* **22**, 1201–1211 (2010). 19413471 10.1162/jocn.2009.21267

[67] Casula, E. P., Rocchi, L., Hannah, R. & Rothwell, J. C. Effects of pulse width, waveform and current direction in the cortex: a combined cTMS-EEG study. *Brain Stimul.* **11**, 1063–1070 (2018). 29709505 10.1016/j.brs.2018.04.015

[68] Casula, E. P. et al. Cerebellar theta burst stimulation modulates the neural activity of interconnected parietal and motor areas. *Sci. Rep.* **6**, 36191 (2016). 27796359 10.1038/srep36191 PMC5086958

[69] Ilmoniemi, R. J. et al. Neuronal responses to magnetic stimulation reveal cortical reactivity and connectivity. *NeuroReport* **8**, 3537–3540 (1997). 9427322 10.1097/00001756-199711100-00024

[70] Tomassini, A. et al. Active movement restores veridical event-timing after tactile adaptation. *J Neurophysiol* **108**, 2092–2100 (2012). 22832572 10.1152/jn.00238.2012

[71] Maldonado Moscoso, P. A., Cicchini, G. M., Arrighi, R. & Burr, D. C. Adaptation to hand-tapping affects sensory processing of numerosity directly: Evidence from reaction times and confidence. *Proc. R. Soc. B: Biol. Sci.* **287**, 20200801 (2020). 10.1098/rspb.2020.0801 PMC7287367 32453983

[72] Kanai, R., Paffen, C. L. E., Hogendoorn, H. & Verstraten, F. A. J. Time dilation in dynamic visual display. *J. Vis.* **6**, 8–8 (2006). 17209745 10.1167/6.12.8

[73] Breska, A. & Ivry, R. B. Double dissociation of single-interval and rhythmic temporal prediction in cerebellar degeneration and Parkinson’s disease. *Proc. Natl. Acad. Sci. U. S. A.* **115**, 12283–12288 (2018). 30425170 10.1073/pnas.1810596115 PMC6275527

[74] Motala, A., Heron, J., Mcgraw, P. V., Roach, N. W. & Whitaker, D. Temporal rate is not a distinct perceptual metric. *Sci. Rep.* **10**, 8654 (2020). 32457383 10.1038/s41598-020-64984-4 PMC7250920

[75] Rammsayer, T. H. & Brandler, S. Aspects of temporal information processing: A dimensional analysis. *Psychol. Res.* **69**, 115–123 (2004). 14758474 10.1007/s00426-003-0164-3

[76] Pooresmaeili, A., Arrighi, R., Biagi, L. & Morrone, M. C. Blood oxygen level-dependent activation of the primary visual cortex predicts size adaptation illusion. *J. Neurosci.* **33**, 15999–16008 (2013). 24089504 10.1523/JNEUROSCI.1770-13.2013 PMC4888977

[77] Hommel, B. Event files: feature binding in and across perception and action. *Trends Cogn. Sci.* **8**(11), 494–500 (2004). 15491903 10.1016/j.tics.2004.08.007

[78] De Kock, R., Zhou, W., Datta, P., Joiner, W. M. & Wiener, M. The role of consciously timed movements in shaping and improving auditory timing. *Proc. R. Soc. B: Biol. Sci.* **290**(1992), 20222060 (2023). 10.1098/rspb.2022.2060 PMC9890119 36722075

[79] Shi, Z., Ganzenmü Ller, S. & Mü Ller, H. J. Reducing bias in auditory duration reproduction by integrating the reproduced signal. *PLoS ONE* **8**, 62065 (2013). 10.1371/journal.pone.0062065 PMC3628943 23614014

[80] De Kock, R., Gladhill, K. A., Ali, M. N., Joiner, W. M. & Wiener, M. How movements shape the perception of time. *Trends Cogn. Sci.* **25**, 950–963 (2021). 34531138 10.1016/j.tics.2021.08.002 PMC9991018

