# Supplementary material for: No aftereffect of motor duration production on auditory duration perception
Source: Sci Rep. 2026 Jun 23;16:28698. doi: 10.1038/s41598-026-59220-4 (PMC13575250; doi:10.1038/s41598-026-59220-4)
Supplement: Supplementary file 1 [file 41598_2026_59220_MOESM1_ESM.docx]

**No aftereffect of motor duration production on auditory duration perception (supplementary material)**

Alessandro Mancari*^1,2^ and Maria Concetta Morrone^1^

^1^ Department of Translational Research on New Technologies in Medicine and Surgery, University of Pisa, Pisa, Italy. ^2^ Department of Psychiatry, University of Oxford, Oxford. *email: [alessandro.mancari@med.unipi.it](mailto:alessandro.mancari@med.unipi.it)

Here we replicate the main results of the original analysis using a nonlinear model of the adaptation effect that does not include a scaling factor as a function of test duration:

$$Aftereffect= A\cdot\log\left( \frac{D}{D_{adapt}} \right)\cdot e^{\frac{{-\log\left( \frac{D}{D_{adapt}} \right)}^{2}}{{2W}^{2}}}+C$$

**Figure S1: Effects of auditory and motor duration adaptation.**

(**a**) Mixed nonlinear (curve) and linear (dashed line) model analysis of auditory adaptation data (showing mean and standard error across subjects). The p-value refers to the linear slope differing from 0. Note the offset between the two models around the adapted duration (vertical midline), which is absent in Figure 2d. (**b**) Histogram of the slopes obtained from the linear fit of each participant’s estimated durations in baseline and auditory adaptation conditions, within the test duration range highlighted in (a). (**c**) Scatter plot of the same data as in (b), showing individual participants and their average with standard errors. A paired-sample t-test found that the slopes differ significantly between the two conditions (t(15) = -2.79, p = .01, d = -0.70, Bayes Factor = 4.19). (**d-f**) Results of the same linear model analysis applied to the motor adaptation data within the range of test duration derived from the curve in (a). No significant difference was found compared to baseline (Bayes Factor = 0.26).

| **Fixed effects** | | |
| --- | --- | --- |
|  | Estimate | Std. Error |
| *A* | 0.12 | 0.04 |
| *W* | 0.70 | 0.06 |
| *C* | 0.01 | 0.02 |
| **Random effects** | | |
| τ_A_ | | 0.03 |
| τ_C_ | | 0.01 |
| ρ_A–C_ | | 0.00 |
| N Subject | | 16 |
| σ² | | 0.01 |
| Marginal R² | | 0.069 |
| Conditional R² | | 0.464 |
| N observations | | 656 |

**Table S1: Results of nonlinear mixed-effects model analysis of auditory adaptation data.** *A* amplitude, *W* width, *C* constant, τ variance of random effects, ρ covariance of random effects, σ^2^ error variance.

| **Fixed effects** | | | | | |
| --- | --- | --- | --- | --- | --- |
|  | Estimate | Std. Error | t-statistic | | p-value |
| (Intercept) | 0.05 | 0.03 | 1.45 | | 0.148 |
| Duration | 0.11 | 0.04 | 2.92 | | **0.004** |
| **Random effects** | | | | | |
| τ_Int_ | | | | 0.02 | |
| τ_Dur_ | | | | 0.02 | |
| ρ_Int_-_Dur_ | | | | 0.01 | |
| N Subject | | | | 16 | |
| σ² | | | | 0.01 | |
| Ordinary R² | | | | 0.542 | |
| Adjusted R² | | | | 0.540 | |
| Conditional R² | | | | 0.764 | |
| N observations | | | | 368 | |

**Table S2: Results of linear mixed-effects model analysis of auditory adaptation data.** τ variance of random effects, ρ covariance of random effects, σ^2^ error variance. Significant effects are in bold.

| **Fixed effects** | | | | | |
| --- | --- | --- | --- | --- | --- |
|  | Estimate | Std. Error | t-statistic | | p-value |
| (Intercept) | -0.01 | 0.03 | -0.31 | | 0.755 |
| Duration | 0.00 | 0.03 | 0.04 | | 0.968 |
| **Random effects** | | | | | |
| τ_Int_ | | | | 0.01 | |
| τ_Dur_ | | | | 0.01 | |
| ρ_Int_-_Dur_ | | | | 0.01 | |
| N Subject | | | | 16 | |
| σ² | | | | 0.01 | |
| Ordinary R² | | | | 0.312 | |
| Adjusted R² | | | | 0.310 | |
| Conditional R² | | | | 0.597 | |
| N observations | | | | 368 | |

**Table S3: Statistical results of linear mixed-effects modelling of motor adaptation data.** τ variance of random effects, ρ covariance of random effects, σ^2^ error variance.
